# High levels of women's satisfaction and compliance with transdermal contraception: results from a European multinational, 6-month study

**DOI:** 10.3109/09513590.2010.538095

**Published:** 2010-12-10

**Authors:** AJ Jakimiuk, PG Crosignani, T Chernev, V Prilepskaya, P Bergmans, M Von Poncet, S Marelli, EJ Lee

**Affiliations:** 1 Center for Reproductive Health, Institute of Mother and Child, Warsaw, Poland; 2 II Institute of Obstetrics and Gynecology, University of Milan, Milan, Italy; 3 Clinic for Fetal Medicine, University Maternity Hospital “Maichin Dom”, Sofia, Bulgaria; 4 Research Centre of Obstetrics, Gynecology and Perinatology, Russian Academy of Medical Sciences, Moscow, Russia; 5 Janssen-Cilag B. V, The Netherlands; 6 Janssen-Cilag, Switzerland; 7 Medical Affairs, Janssen-Cilag, Italy; 8 EMEA Medical Affairs, Janssen-Cilag EMEA, Belgium

**Keywords:** Transdermal patch, hormonal contraception, compliance, satisfaction, preference

## Abstract

**Objective:**

To investigate compliance, satisfaction, and preference in women using a transdermal contraceptive patch.

**Methods:**

Women (18–6 years) from eight European countries used contraceptive patches (norelgestromin 6 mg, ethinylestradiol 600 μg for six, 4-week treatment cycles. Compliance, satisfaction, and preference were assessed after 3 and 6 cycles and study completion using self-report methods.

**Results:**

Of the 778 participants, 36.8% (*n* = 287) used no contraception at baseline. The most common methods were oral contraceptives (67.9%, *n* = 334) and barrier methods (21.5%, *n* = 106). Of oral contraception users, 63.5% (*n* = 212) were satisfied or very satisfied with their previous method, but compliance was poor with 77.8% (*n* = 260) reporting missed doses. After 3 and 6 cycles, > 80% of all included women were satisfied or very satisfied with the patch. At study completion, most participants (73.7%) reported a preference for the patch compared to their previous method. Of 4107 cycles, 3718 (90.5%) were completed with perfect compliance. Two pregnancies occurred during this study, representing a Pearl Index of 0.63. No new safety issues were identified and the patch was well tolerated.

**Conclusion:**

Women were highly satisfied with transdermal contraception and preferred this form of family planning over their previous method. Transdermal contraception represents a valuable addition to contraceptive options with potential to offer high compliance and efficacy.

## Introduction

Compliance and correct use of contraceptives is of critical importance in the prevention of pregnancy. Minimizing the frequency of dosing is one way of improving adherence with hormonal contraception. Some nonhormonal contraceptives have been introduced to reduce the risk of “missing pills,” e.g., the transdermal patch and the vaginal ring. The first and only transdermal contraceptive patch to receive widespread regulatory approval, EVRA®/ORTHO EVRA® (ethinylestradiol/norelgestromin), is a convenient approach to contraception that has a similar efficacy to oral contraceptives, but with the benefit of once-weekly administration. In addition, transdermal delivery of contraceptive hormones provides a number of differences compared to the oral route: elimination of the variability in gastrointestinal absorption, avoidance of the hepatic first-pass metabolism, and prevention of peaks and troughs in serum concentrations that are seen with oral contraceptives [1,2].

The efficacy of the contraceptive patch has been clearly demonstrated in three Phase III studies; two of which were randomized comparisons with an oral contraceptive. The safety and tolerability profile of the patch was shown to be comparable to that of oral contraceptives [1,3-10].

Compliance as well as satisfaction with the contraceptive patch has been shown to be significantly better than with oral contraceptives in a clinical trial setting [4,11]. In a large clinical trial, North American women of all ages (18-45 years) were able to use the patch more consistently and correctly, than the comparative oral contraception users [12]. Another, more recent, comparative study that was conducted in Europe and South Africa, found that, overall, patch users had significantly more cycles with perfect compliance than oral contraception users (*P* < 0.001) [13]. The patch users also reported a high level of satisfaction and improvement in emotional and physical well-being, compared to the oral contraception group [11,13].

The dosing regimen of the transdermal contraceptive patch mimics that of most oral contraceptives: 21 days of active hormones, followed by 7 days without hormones, but with the advantage that the patch only requires a once-a-week application for three consecutive weeks, followed by one patch-free week. The patch may be applied to the upper outer arm, lower abdomen, upper torso, or buttocks [14].

Results from three clinical trials in more than 3000 women have shown that efficacy and cycle control are similar with transdermal contraception to those reported for oral contraceptives [3]. Early studies in women aged 18-45 years have shown that compliance with the weekly patch is significantly better than with a daily oral contraceptive dosing regimen across all cycles and all age groups [12,15]. In contrast, compliance with the oral contraceptive was significantly related to age, ranging from 68% in younger women aged less than 20 years, to 85% in women aged 40-45 years [12]. It is hypothesized that the improved compliance with the patch could result in fewer unintended pregnancies compared to an oral contraceptive in a large population [16].

As human behavior tends to be reinforced when rewarded, satisfaction with a contraceptive method is essential for long-term adherence, and depends mainly on selecting the optimal contraceptive method [2].

In this context, satisfaction encompasses a number of factors relating to the user's experience, including ease of use, acceptance by self and partner, tolerability, and effectiveness. Women who are satisfied with their chosen method of contraception are more likely to be compliant with the regimen, hence optimizing contraceptive efficacy.

The main objective of this pan-European study was to evaluate women's experience with transdermal contraception, focusing on compliance, satisfaction, and user preference. The results of the Italian subgroup of this study were already reported in 2009 [17]. This article provides an overview of the results of the entire population that participated in the study.

## Methods

### Study design

This open-label, single-arm study was conducted in Belgium, Bulgaria, France, Ireland, Italy, Poland, Romania, and Russia. Participating women received contraception via a patch (EVRA®, Janssen-Cilag) for 24 weeks, comprising six, 4-week treatment cycles.

The study was conducted in accordance with the Declaration of Helsinki and subsequent revisions, local ethics committee approval was obtained and all participants provided written informed consent.

### Participants

Participants were women aged 18–46 years who had a regular menstrual cycle occurring every 25–35 days (unless using an implant, which was removed immediately before the application of the patch. In case of injectable contraceptives, the patch was applied 12 weeks – 12 weeks and 5 days after the last injection), were sexually active, and at risk of pregnancy. Participants were required to have a normal Pap smear within the 12 months before enrolment and a negative urine pregnancy test at the screening visit, with confirmation of start of menses before application of the first patch. The main exclusion criteria for the study were as follows: known contraindications to hormonal contraceptives; recent history of alcohol or substance abuse; skin conditions associated with oily, irritated or damaged skin at all potential application sites; and chronic use of barbiturates, antiepileptics, rifampin, griseofulvin, or other hepatic enzyme-inducing agents or systemic antibiotics.

### Study medication

The 20 cm^2^ patch contains norelgestromin 6 μg and ethinylestradiol 600 μg. During each cycle, a patch was worn for 1 week and replaced on the same day of the week for three consecutive weeks. The patch could be placed on the buttocks, abdomen, upper torso (with the exception of the breasts), or the upper outer arm. The fourth week of each cycle was patch-free.

### Assessments

Participants visited the clinic for assessment at screening (Visit 1), and at the end of Cycles 1 (Visit 2), 3 (Visit 3), and 6 (Visit 4). Individuals were also contacted by telephone on Day 1 of Cycle 1.

At screening, participants who were currently using a method of contraception completed a standard set of questions regarding their satisfaction with their method. At the end of Cycles 3 and 6, participants completed the same standard set of questions regarding their satisfaction with the patch. In addition, at the end of Cycle 6 (or on early withdrawal), participants were asked to compare the patch with their previous method, if applicable, and about their future contraceptive choice.

Satisfaction was assessed by a number of questions, each evaluated on a 5-point scale. Questions included: (1) Overall satisfaction (very dissatisfied, dissatisfied, neither satisfied nor dissatisfied, satisfied, very satisfied); (2) Convenience (very inconvenient, inconvenient, neither convenient nor inconvenient, convenient, very convenient), (3) Worry about getting pregnant (all of the time, most of the time, some of the time, a little of the time, none of the time), and (4) Satisfaction with adhesion of the patch (very dissatisfied, dissatisfied, neither satisfied nor dissatisfied, satisfied, very satisfied). Women were also asked to state how strongly they agreed or disagreed with a number of statements, again assessed on a 5-point scale (strongly disagree, disagree, neither agree nor disagree, agree, strongly agree): I easily incorporated the method into my lifestyle; I am satisfied with the frequency - once a week; I feel secure that the patch works; The patch has improved my sex life; My partner accepts the patch.

Quality of life was assessed at screening and at the end of Cycles 3 and 6 using the validated Short Form 12 (SF-12) instrument [18]. Compliance was assessed by diary cards. Perfect compliance was defined on the basis of five criteria: first patch to be applied on the first day of menses; patch to be worn for not more than 7 days; continuous patch use for 21 days; no interval between successive patches; a patch-free interval of 1-7 days in the 4th week of the cycle.

Safety was assessed at each postscreening contact (i.e., study visits and telephone contact), and investigators assessed any possible relationship to the contraceptive patch. Urine pregnancy tests were carried out at screening, at the end of the study, and at any visit if pregnancy was suspected.

Efficacy was assessed using the Pearl index (i.e., the number of pregnancies per 100 woman-years of use).

### Analysis

The primary aims of the study were to assess the level of satisfaction with the patch and, where applicable, to compare satisfaction with the patch with the subject's previous contraceptive method.

As the results were to be reported descriptively, no formal sample size calculation was performed. It was expected that the inclusion of approximately 1000 subjects would be sufficient to allow for various subgroup analyses.

Demographic variables, obstetric and gynecological history, primary contraceptive information at baseline, and endpoint parameters were tabulated. Between-group comparisons were made using the Wilcoxon two-sample test for ordinal and interval variables and Fisher's exact test for nominal variables. Within-group comparisons of shifts versus baseline were made using the Wilcoxon signed rank test. All statistical comparisons were made using two-tailed analyses with *P* ≤ 0.05 required for significance.

The statistical analyses were performed using SAS version 9.1 (SAS Institute, 1999–2001). The SF-12 (version 1) physical component summary (PCS) and mental component summary (MCS) were calculated according to Ware [19].

## Results

### Participants

From the eight European countries involved, 105 active sites participated to the trial (40 in France, 23 in Italy, 11 in Romania, 9 in Belgium, 8 in Poland, 7 in Ireland, 4 in Bulgaria, and 3 in Russia), and in total, 820 women were screened for the study. Of these, 38 did not receive study treatment and were therefore not included in the analysis. Two other participants were lost to follow-up after Visit 1 (it was not clear whether they even used the patch at all). Of the 780 women who received at least one patch (207 in Italy, 147 in France, 103 in Romania, 100 in Poland, 86 in Belgium, 61 in Bulgaria, 58 in Russia, and 18 in Ireland), two did not perform a final visit, meaning that endpoint data were available for 778 participants. Baseline characteristics are shown in Table I. Almost half of the women had previously been using oral contraception. The study was completed by 642 (82.3%) of the 780 women who received at least one transdermal patch. The most common reasons for withdrawal from the study were AEs (*n* = 55, 39.9%), withdrawal of consent (*n* = 31, 22.5%), and lost to follow-up (*n* = 22, 15.9%).

**Table I:** Baseline characteristics.

	*N* = 780 mean ± SD, unless stated
Age, years	28.4 ± 6.2
Height, cm	165.0 ± 6.1
Weight, kg	58.9 ± 9.2
BMI < 30 kg/m^2^, *n* (%)	763 (97.8)
BMI ≥ 30kg/m^2^, *n* (%)	17 (2.2)
Pregnancy history, *n* (%)	
0	358 (45.9)
1	168 (21.5)
2	151 (19.4)
>2	103 (13.2)
Delivery history, *n* (%)	
0	430 (55.1)
1	184 (23.6)
2	139 (17.8)
>2	27 (3.5)
Contraception used in the previous 3 months, *n* (%)	
None	243 (31.2)
Oral	354 (45.4)
Barrier	133 (17.1)
Other*	50 (6.4)
Mean duration of previous contraceptive use, days	861.6 ± 1208

BMI, body mass index.

* Includes intrauterine device, implants, injection, withdrawal, fertility awareness, vaginal ring, spermicide, postcoital contraception (emergency contraception), and abstention.

### Satisfaction with and preference of contraceptive method

**Figure 1:**
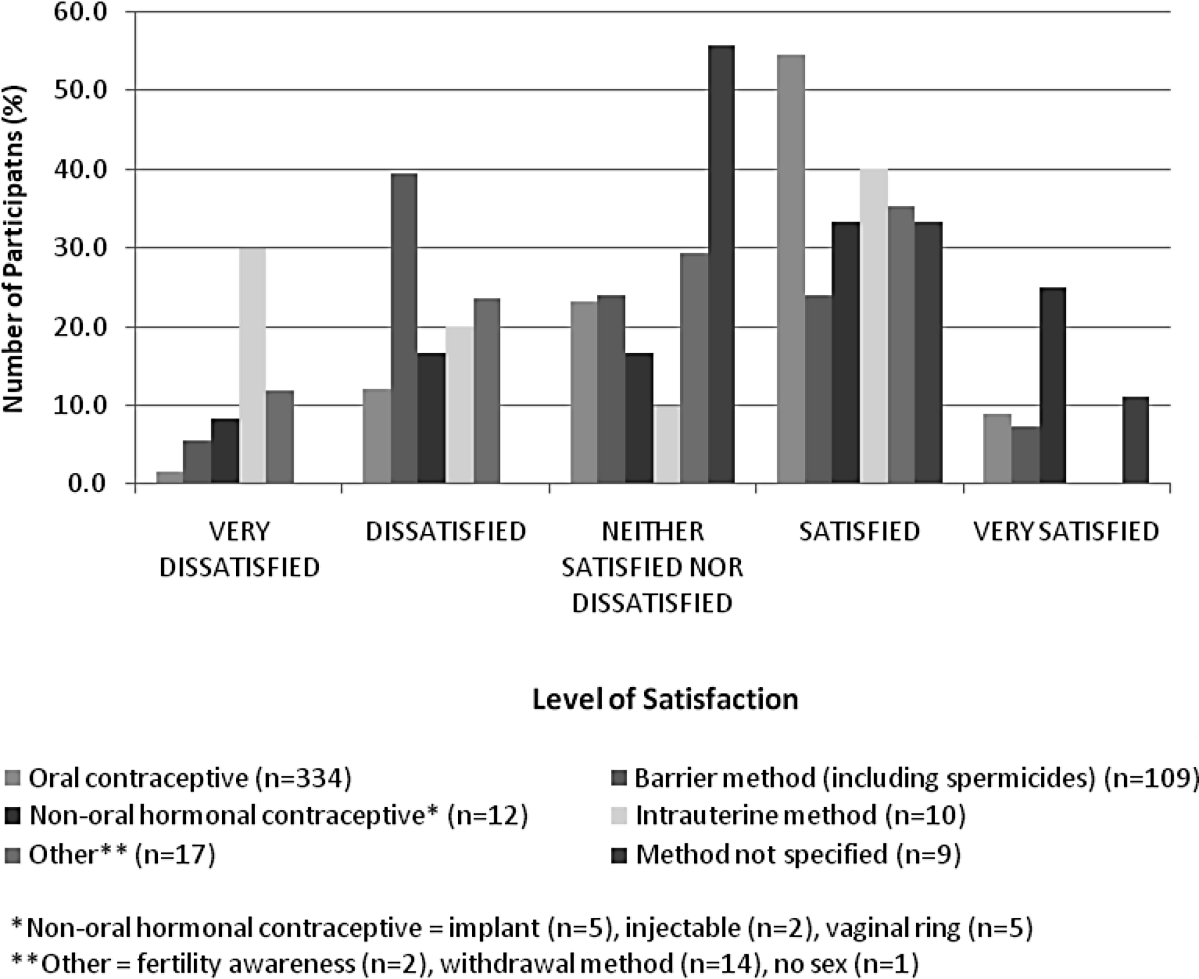
Overall satisfaction with contraception used at baseline.

For the women who were using a contraceptive method at baseline (*n* = 482), overall satisfaction at baseline is shown in Figure 1. Women were generally more satisfied with oral contraception (63.5% of users) than with barrier methods (31.2% of users).

**Figure 2:**
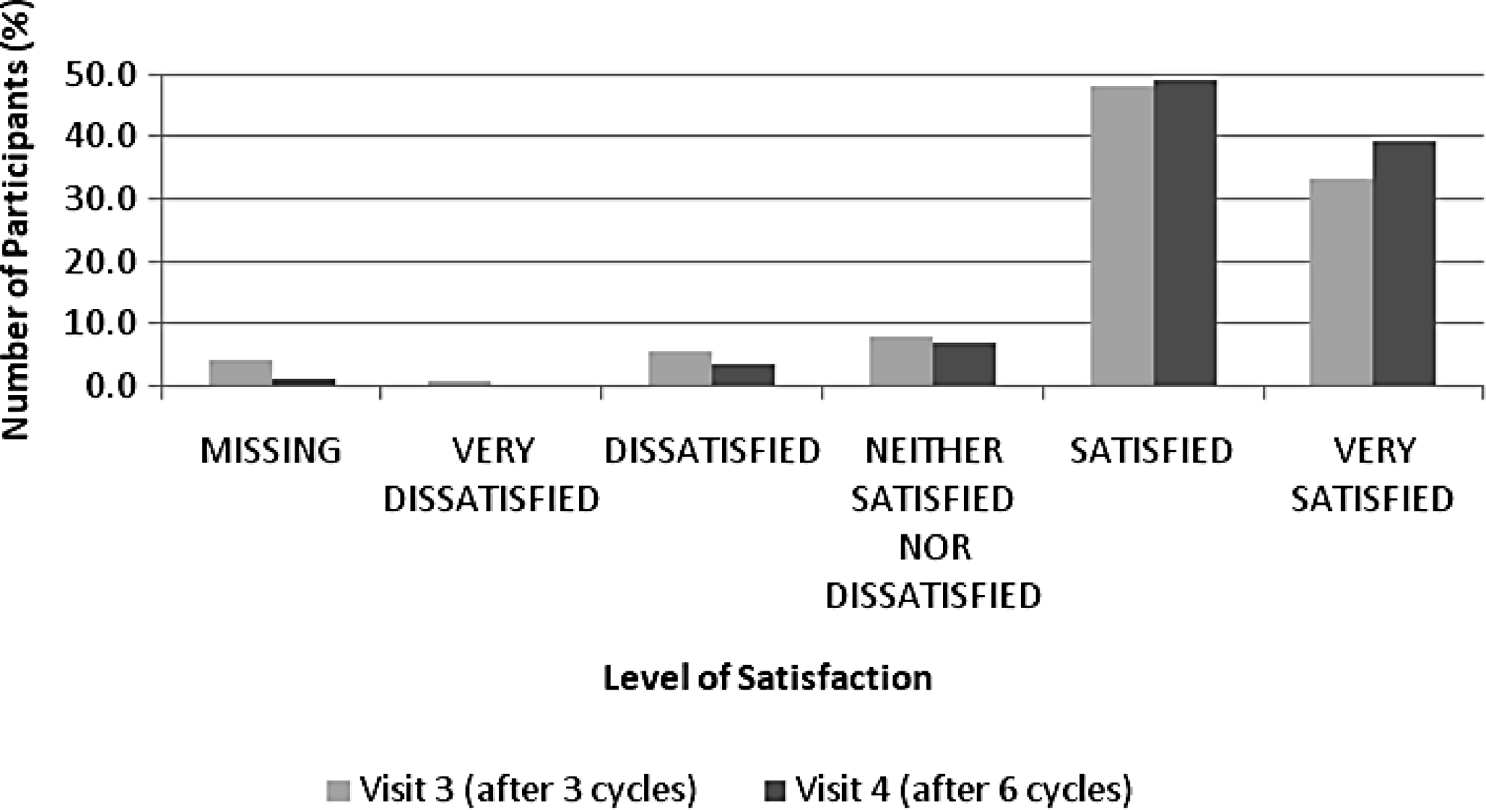
Overall satisfaction with the patch after 3 (Visit 3) and 6 cycles (Visit 4) in the whole study group.

Overall satisfaction with transdermal contraception in the whole study group is shown in Figure 2.

**Figure 3:**
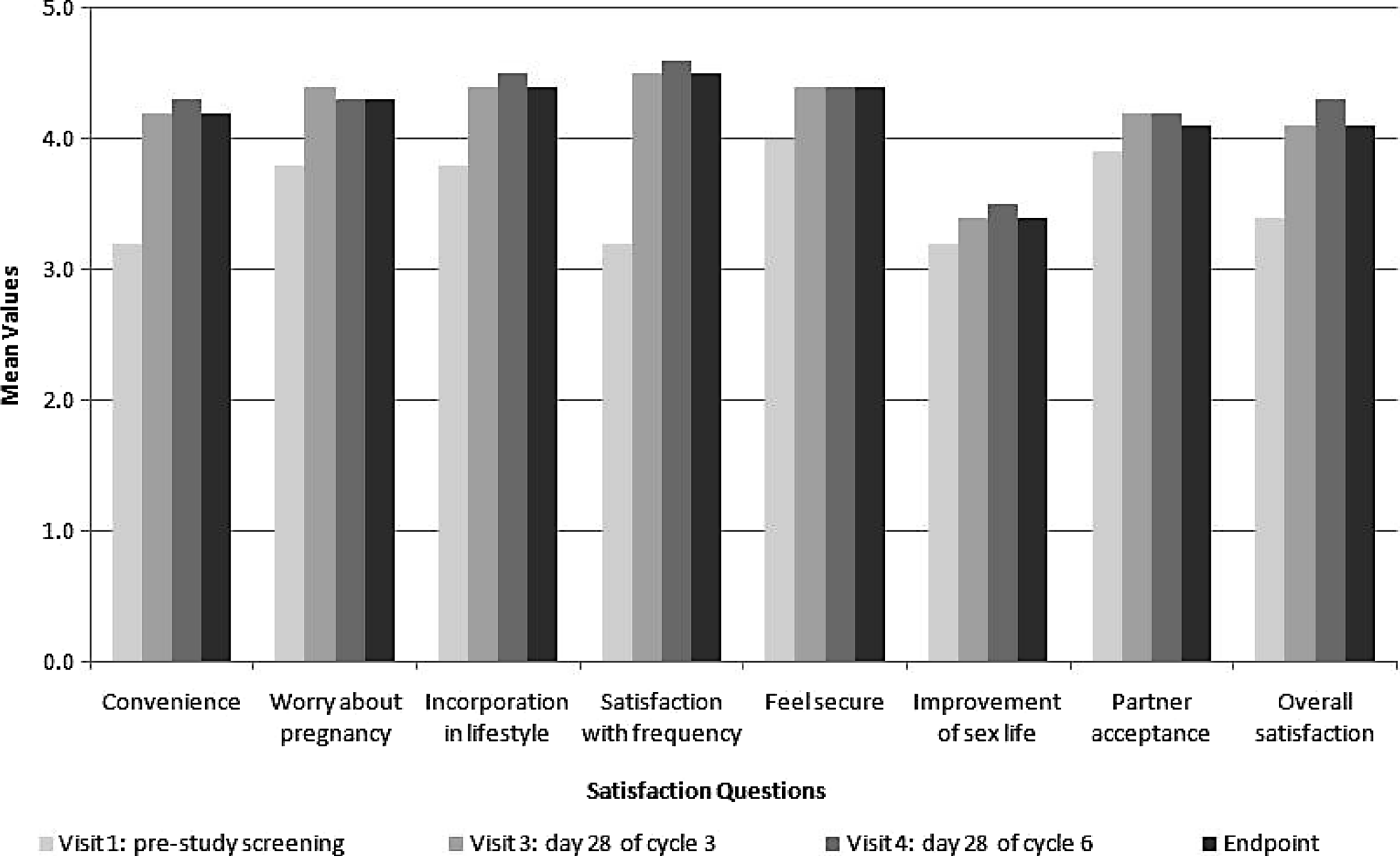
Satisfaction questions: mean scores at each visit for the subset of women currently using a contraceptive method at baseline.

In general, women who were using a contraceptive method at baseline reported higher satisfaction with the patch than with their previous contraceptive method. At baseline, 54.4% of women using contraception were satisfied or very satisfied with their chosen method. Compared to the start of the study, after three cycles (Visit 3), 83.0% of these women were satisfied or very satisfied with the patch, and after six cycles (Visit 4), the percentage increased to 87.5%. Considering the subset of women currently using a contraceptive method at baseline, the largest change was seen with regard to the participants’ satisfaction with the once-a-week frequency of patch application (Figure 3), which improved by 1.4 points on a 5-point scale compared with their previous contraceptive method (*P* = 0.0001). There was no significant change in participants’ response to the question about improvement in their sexual life or the partner's acceptance of the patch.

Table II presents the most prominent improvements compared to baseline, seen on individual questions relating to convenience, worrying about pregnancy, ease of incorporation into lifestyle, satisfaction with the once-a-week frequency, feeling of security, and overall satisfaction of the patch. This table compares the patch evaluation of the whole study group (including naive women to contraceptive methods) versus women currently using a method at baseline.

**Table II:** Significant improvements on individual questions.

	Baseline (%)	Visit 3 (%)	Visit 4 (%)
			
	(Prev. method)	(Patch)	(Patch)
	*n* = 491	*n* = 759	*n* = 659
(Very) Convenient	45.5	83.1	89.2
Worry about pregnancy: all/most of the time	10.6	4.6	3.4
(Very) Easily incorporated into lifestyle	72.7	89.0	93.8
(Very) Satisfied with frequency of dosing	44.3	91.3	95.7
Feel (very) secure	79.4	85.9	92.3
Overall (very) satisfied	54.4	81.3	88.2

The percentages at baseline are referred to women currently using a contraception method; percentage at Visits 3 and 4 are referred to the whole study group.

*n* = number of subjects at the specified visit.

Two-sided Wilcoxon signed rank test.

Considering the subset of women currently using a contraceptive method at baseline, 87% of participants reported they were satisfied or very satisfied regarding the adhesion of the patch at the end of Cycle 6. At the end of the last cycle, almost 90% recorded that the patch was convenient or very convenient, 92% felt secure that the patched worked, and 81% (strongly) agreed that their partner accepted the patch.

The questions for Visit 1 are referred to the method used at baseline, for the other visits they are referred to the patch.

**Figure 4:**
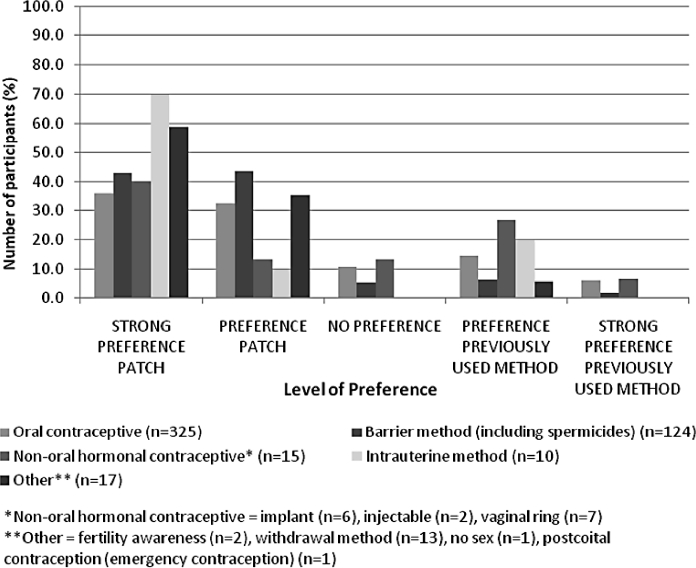
Personal preference between the patch and the previous method. Previous method means either the method currently used at baseline or that had been used in the 3 months prior to study entry.

Most participants using contraception at baseline or having used an anticonceptive method in the 3 months prior to study start, reported a preference for the patch over their previous method (Figure 4), regardless of the type of contraception; 68.6% of women using oral contraceptives and 86.2% using barrier methods at baseline reported a preference or strong preference for the patch. The reasons most often cited for the preference of the patch were convenience (45.4%), ease of use/simplicity (28.8%) and fewer side effects (17.5%).

Of the women who were not using a contraceptive method at baseline of the study (*n* = 287), 83.5% and 90.7% also were satisfied or very satisfied with the patch at the end of Visits 3 and 4, respectively. After three cycles, 83.9% found the patch convenient or very convenient, 88.6% incorporated the method easily into their lifestyle, and 83.2% felt secure that the patch worked. After six cycles, these percentages increased to more than 90% for these aspects. This satisfaction was reflected to a lesser extent in the choice for the future method because 48.0% indicated they would use the patch in future, 14.3% said they would use oral contraceptives and 22.0% said they would not use any contraceptive method in the future. Overall, 73.7% of the participants in the entire study group had a preference for the patch compared to their previous contraceptive method at the final visit, and if given the choice, they would also continue using this method of contraception.

### Compliance

At baseline, self-reported compliance with oral contraceptives was poor. Only 22.2% of women stated that they never forgot to take their contraception, with 35.9% reporting they forgot to use their contraceptive “some,” “most” or “all of the time.” Just over half of oral contraceptive users (52.5%) considered this to be at least a convenient method (42.0% convenient; 10.5% very convenient).

Based on participants’ diaries, 3718 of 4107 recorded cycles (90.5%) were completed with perfect compliance with patch use. Despite the stringency of the criteria for perfect compliance, 67.7% of participants were fully compliant with all their cycles. The most common reason for not achieving perfect compliance was that a patch was not on the skin for exactly 21 consecutive days (6.0%). The mean compliance score (calculated for each individual as the number of cycles with perfect compliance divided by the total number of cycles) was 86.2% (±26.4 SD). The main reasons for patch removal were scheduled change (93.0%), patch partially lifted off (3.3%) and patch fell off (2.9%). Four women (2.9%) were withdrawn from the study as a result of poor compliance.

### Quality of life

Mean scores on the Physical Component Summary (PCS) of the SF-12 remained unchanged throughout the study (baseline, 54.0 ± 4.7 SD; end of Cycle 6, 54.2 ± 4.3 SD). Scores on the Mental Component Summary (MCS) increased from 50.5 ± 7.8 at baseline to 52.0 ± 7.2 SD at the end of Cycle 3 and 52.4 ± 7.3 SD at the end of Cycle 6. No statistically significant changes in PCS were observed, compared to baseline. However, the mean changes from baseline to the end of Cycles 3 and 6 in MCS were statistically significant (*P* = 0.0001, in both cases), but these small changes were unlikely to have been of meaningful clinical relevance.

### Safety

During the 24-week study period, treatment emergent adverse events (AEs) were reported by 42.3% of participants. During the first cycle, 57.9% of these AEs started and most (45.6%) of these AEs also stopped during this cycle. The frequency of occurrence tended to decrease over time; 12.8% of women reported AEs in the 3rd cycle, whereas 6.9% in the 6th cycle. The most common AEs were spotting between menses (9.0% of participants), breast pain (8.2%) and headache (6.5%). Most AEs were considered mild (52.9%) or moderate (36.2%) in severity and were not or doubtfully (35.0%), possibly (23.0%), probably (20.4%), or very likely (21.7%) related to the use of the transdermal patch. The application of the patch was permanently stopped in 12.8% of AEs. Most AEs (90.4%) were resolved before the end of the study. Fifty-five (7.1%) participants dropped-out of the study due to an AE.

Three women experienced a serious adverse event (SAE) during the study, which required hospitalization (radiculitis, bartholinitis, and aggravated Graves-Basedow disease with increased exophthalmia and diplopia); however, none of them were considered related to the transdermal patch, and all participants recovered from these SAEs.

No clinically relevant deviations were observed regarding weight change or vital signs.

At baseline, participants mean weight was 58.9 kg (±9.2 kg SD).

At Cycle 6, a mean weight increase of 0.4 kg (±2.5 kg SD) versus baseline was observed (*P* = 0.0001), representing a mean increase of 0.8% (±4.2% SD). Women aged less than 21 years, 21-25 years, and 31-40 years, experienced a mean weight gain of 0.5-0.6 kg, whereas women aged 26-30 years, and 41-50 years, only gained 0-0.1 kg. No clear differences in weight were seen between participants who previously used contraception and those who did not.

At baseline, the participant's mean BMI was 21.6 kg/m^2^ (±3.2 kg/m^2^ SD). At Cycle 6, a mean BMI increase of 0.2 kg/m^2^ (±0.9 kg/m^2^ SD) versus baseline was noted (*P* = 0.0001), also representing a mean increase of 0.8% (±4.0% SD).

No statistically significant differences from baseline were reported regarding pulse, systolic blood pressure, and diastolic blood pressure.

### Efficacy

Two pregnancies occurred during this study, representing a Pearl index (estimate of the number of pregnancies per 100 woman-years of use) of 0.63.

## Discussion

It is widely accepted that compliance, and thus efficacy, is generally higher in clinical studies than it is in the ‘real world’ setting. Compliance is, however, likely to be increased if individuals are highly satisfied with their chosen method of contraception. In this context, our data showing that women switching to the patch were more likely to say they were “satisfied” or “very satisfied” with this contraceptive method are of interest. Women using oral contraceptives at baseline tended to be satisfied with this method, but even amongst this group, over two-thirds of participants expressed a preference for the patch by the end of the 24-week study period. A motivating factor for women to enter the study was likely to have been some level of dissatisfaction with their current method of contraception, thus some improvement in satisfaction scores was to be expected.

The convenience and ease of use of the patch were cited as the most important factors contributing to preference over previous forms of contraception. Interestingly, almost 20% of participants cited fewer AEs as their reason for preferring the patch. The high level of subject satisfaction from patient questionnaires was accompanied by positive findings in the SF-12 Quality of Life (QoL) instrument. While the mean scores on the PCS of the SF-12 remained unchanged throughout the study, statistically significant changes in the mean MCS scores were observed at the end of Cycles 3 and 6. However, these changes were small and unlikely to have meaningful impact on overall QoL [19]. Given the study population comprised generally healthy women with already robust baseline QoL scores, satisfaction in contraception choice is unlikely to shift overall QoL. Nevertheless, the improved satisfaction, and preference of the patch over previous method indicates a generally positive experience for women using the contraceptive patch and this should not be ignored as an important factor in women's physical and mental health and well-being.

The data obtained in our study are consistent with those from previous studies of participant satisfaction and well-being while using the contraceptive patch. Significantly more patch users were very satisfied with their study regimen compared with the oral group, and there was a weak correlation between overall satisfaction and both mental and physical well-being [13]. Data from our study were obtained from eight European countries, while those of the previously cited were from 65 centers in Europe and South Africa, indicating that the results are not necessarily culturally specific. Furthermore, similar results have been reported recently in a Canadian trial, in which 91% of those who completed the nine-cycle study were satisfied with the patch, and about 75% of those who completed all nine cycles expressed a preference for the patch over their previous contraception [20].

As might be expected from the high level of satisfaction with treatment, compliance in the study was high (90.5%). This supports other findings that have shown transdermal contraception to be associated with a significantly greater proportion of perfect cycle compliance than oral contraceptives [13,16,21,22]. One of the main factors known to affect compliance is the inconvenience of frequent dosing regimens. Indeed, reductions in dose frequency have been shown to improve compliance in a number of therapeutic areas, leading to improvements in outcomes [23]. In a meta-analysis conducted by the Cochrane Collaboration of studies involving the contraceptive patch, the authors reported that compliance was significantly greater with the weekly patch than with daily oral contraception (odds ratio: 2.1; 95% Confidence Interval: 1.8-2.3) [24].

Patch contraception was generally well tolerated in our study, which likely contributed to the high levels of satisfaction and compliance. Although more than 40% of participants reported AEs, most events started and stopped during the first cycle. It should also be noted that AEs tended to be more prevalent in early cycles, which is consistent with findings in previous studies. During the study, three women experienced SAEs which required hospitalization (radiculitis, bartholinitis, and aggravated Graves-Basedow disease with increased exophthalmia and diplopia); however, none of them were considered related to the transdermal patch, and all participants recovered from these SAEs. Two pregnancies occurred during this study, yielding a Pearl index of 0.63, which is consistent with previous studies [3,15,22].

In conclusion, women generally have a high level of satisfaction with transdermal contraception and expressed a preference for this form of family planning over their previous method. This method thus represents a valuable addition to contraceptive options with the potential to offer high compliance and thus efficacy.

## References

[1] Graziottin A (2006). A review of transdermal hormonal contraception. Focus on the ethinylestradiol/norelgestromin contraceptive patch. Treat Endocrinol.

[2] Graziottin A (2008). Safety, efficacy and patient acceptability of the combined estrogen and progestin transdermal contraceptive patch: a review. Patient Prefer Adherence.

[3] Zieman M, Guillebaud J, Weisberg E, Shangold GA, Fisher AC, Creasy GW (2002). Contraceptive efficacy and cycle control with the Ortho Evra/Evra transdermal system: the analysis of pooled data. Fertil Steril.

[4] Burkman RT (2007). Transdermal hormonal contraception: benefits and risks. Am J Obstet Gynecol.

[5] Jick SS, Kaye JA, Russmann S, Jick H (2006). Risk of nonfatal venous thromboembolism in women using a contraceptive transdermal patch and oral contraceptives containing norgestimate and 35 μg of ethyl estradiol. Contraception.

[6] Jick S, Kaye JA, Li L, Jick H (2007). Further results on the risk of nonfatal venous thromboembolism in users of the contraceptive transdermal patch compared to users of oral contraceptives containing norgestimate and 35 μg of ethinyl estradiol. Contraception.

[7] Cole JA, Norman H, Doherty M, Walker AM (2007). Venous thromboembolism, myocardial infarction, and stroke among transdermal contraceptive system users. Obstet Gynecol.

[8] Jick SS, Hagberg KW, Hernandez RK, Kaye JA (2010). Postmarketing study of ORTHO EVRA® and levonogestrel oral contraceptives containing hormonal contraceptives with 30 mcg of ethinyl estradiol in relation to nonfatal venous thromboembolism. Contraception.

[9] Dore DD, Norman H, Seeger JD (2009). Eligibility criteria in venous thromboembolism, myocardial infarction, and stroke among transdermal contraceptive users. Letter to the editor of Obstet Gynecol.

[10] Dore DD, Norman H, Loughlin J, Seeger JD (2010). Extended case-control study results on thromboembolic outcomes among transdermal contraceptive users. Contraception.

[11] Wan GJ, Barnowski CE, Ambegaonkar BM, Bolge SC, McDonnell DD (2007). Treatment satisfaction with a transdermal contraceptive patch or oral contraceptives. Contraception.

[12] Archer DF, Bigrigg A, Smallwood GH, Shangold GA, Creasy GW, Fisher AC (2002). Assessment of compliance with a weekly contraceptive patch (Ortho Evra™/Evra™) among North American Women. Fertil Steril.

[13] Urdl W, Apter D, Alperstein A, Koll P, Schonian S, Bringer J, Fisher AC, Preik M (2005). Contraceptive efficacy, compliance and beyond: factors related to satisfaction with once-weekly transdermal compared with oral contraception. Eur J Obstet Gynecol Reprod Biol.

[14] Lopez LM, Grimes DA, Gallo MF, Schulz KF (2008). Skin patch and vaginal ring versus combined oral contraceptives for contraception (Review). The Cochrane Collaboration.

[15] Audet MC, Moreau M, Koltun WD, Waldbaum AS, Shangold G, Fisher AC, Creasy GW (2001). Evaluation of contraceptive efficacy and cycle control of a transdermal contraceptive patch vs. an oral contraceptive: a randomized controlled trial. JAMA.

[16] Archer DF, Cullins V, Creasy GW, Fisher AC (2004). The impact of improved compliance with a weekly contraceptive transdermal system (Ortho Evra) on contraceptive efficacy. Contraception.

[17] Crosignani PG, Nappi C, Ronsini S, Bruni V, Marelli S, Sonnino D, EVRA Contrast Study Group (2009). Satisfaction and compliance in hormonal contraception: the result of a Multicentre Clinical Study on Women's Experience with the ethinylestradiol/norelgestromin contraceptive patch in Italy. BMC Woman's Health.

[18] Ware J, Kosinski M, Keller SD (1996). A 12-Item Short-Form Health Survey: construction of scales and preliminary tests of reliability and validity. Med Care.

[19] Ware JE, Kosinski M, Turner-Bowker DM, Gandek B (2004). How to score Version 2 of the SF-12 Health Survey (with a supplement documenting version 1).

[20] Weisberg F, Bouchard C, Moreau M, Audet MC, Mawdsley S, Dattani D, Dinniwell J, Horbay GL (2005). Preference for and satisfaction of Canadian women with the transdermal contraceptive patch versus previous contraceptive method: an open-label, multicentre study. J Obstet Gynaecol Can.

[21] Creasy GW, Abrams LS, Fisher AC (2001). Transdermal contra ception. Semin Reprod Med.

[22] Smallwood GH, Meador ML, Lenihan JP, Shangold GA, Fisher AC, Creasy GW (2001). Efficacy and safety of a transdermal contraceptive system. Obstet Gynecol.

[23] Richter A, Anton SE, Koch P, Dennett SL (2003). The impact of reducing dose frequency on health outcomes. Clin Ther.

[24] Gallo MF, Grimes DA, Schulz KF (2003). Skin patch and vaginal ring versus combined oral contraceptives for contraception. Cochrane Database Syst Rev.

